# Determination of lumefantrine as an effective drug against *Toxoplasma gondii* infection – *in vitro* and *in vivo* study

**DOI:** 10.1017/S0031182020002036

**Published:** 2021-01

**Authors:** Dawei Wang, Mengen Xing, Saeed El-Ashram, Yingying Ding, Xiao Zhang, Xiaoyu Sang, Ying Feng, Ran Chen, Xinyi Wang, Ning Jiang, Qijun Chen, Na Yang

**Affiliations:** 1 Key Laboratory of Livestock Infectious Diseases in Northeast China, Ministry of Education, Key Laboratory of Zoonosis, College of Animal Science and Veterinary Medicine, Shenyang Agricultural University, Dongling Road 120, 110866 Shengyang, China; 2 College of Food Science, Shenyang Agricultural University, Dongling Road 120, 110866 Shengyang, China; 3 College of Life Science and Engineering, Foshan University, 18 Jiangwan Street, Foshan, 528231, Guangdong Province, China; 4 Faculty of Science, Kafrelsheikh University, Kafr El-Sheikh, 33516, Egypt; 5 Department of Preventive Veterinary Medicine, College of Veterinary Medicine, Shandong Agricultural University, Taian City, Shandong Province, China

**Keywords:** Anti-*Toxoplasma gondii*, lumefantrine, proliferation, *Toxoplasma gondii*, treatment

## Abstract

*Toxoplasma gondii* is an obligate intracellular protozoan parasite, which can infect almost all warm-blooded animals, including humans, leading to toxoplasmosis. Currently, the effective treatment for human toxoplasmosis is the combination of sulphadiazine and pyrimethamine. However, both drugs have serious side-effects and toxicity in the host. Therefore, there is an urgent need for the discovery of new anti-*T. gondii* drugs with high potency and less or no side-effects. Our findings suggest that lumefantrine exerts activity against *T. gondii* by inhibiting its proliferation in Vero cells *in vitro* without being toxic to Vero cells (*P* ≤ 0.01). Lumefantrine prolonged mice infected with *T. gondii* from death for 3 days at the concentration of 50 *μ*g L^−1^ than negative control (phosphate-buffered saline treated only), and reduced the parasite burden in mouse tissues *in vivo* (*P* ≤ 0.01; *P* ≤ 0.05). In addition, a significant increase in interferon gamma (IFN-γ) production was observed in high-dose lumefantrine-treated mice (*P* ≤ 0.01), whereas interleukin 10 (IL-10) and IL-4 levels increased in low-dose lumefantrine-treated mice (*P* ≤ 0.01). The results demonstrated that lumefantrine may be a promising agent to treat toxoplasmosis, and more experiments on the protective mechanism of lumefantrine should be undertaken in further studies.

## Introduction

*Toxoplasma gondii* is an obligate intracellular protozoan parasite, which can infect almost all warm-blooded, including humans, leading to toxoplasmosis (Dubey, 2010; El-Ashram *et al*., 2015*a*, 2015*b*; Yin *et al*., 2015*a*, 2015b). Approximately 30% of the world's population has serological evidence of *T. gondii* infection (Zhou *et al*., 2011). Toxoplasmosis is normally innocuous in individuals with a good immune system; however, *T. gondii* infection is severe or even fatal for immunocompromised patients, such as those with AIDS, tumour and organ transplant recipients (Tian *et al*., 2012; Qin *et al*., 2014; Wang *et al*., 2016).

Several anti-*T. gondii* drugs, including sulphonamides and pyrimethamine have been used to control toxoplasmosis (Montoya and Liesenfeld, 2004; Meneceur *et al*., 2008; Doliwa *et al*., 2013*a*, 2013*b*). Both sulphonamides and pyrimethamine prevent the synthesis of folate by inhibiting the dihydrofolate reductase and dihydropteroate synthase that are essential for the survival and multiplication of parasites (Derouin, 2001; Anderson, 2005). However, these drugs cannot completely inactivate encysted bradyzoites or treat congenital toxoplasmosis, and their use is also limited by their side-effects, including haematological toxicity (pyrimethamine), cutaneous rash, leucopoenia and thrombocytopoenia (sulphonamides) (Agha *et al*., 1992; Subauste and Remington, 1993; Kim *et al*., 2007; Torre *et al*., 2011). There is increasing evidence of treatment failures in patients affected by toxoplasmosis suggesting the existence of drug resistance in clinical therapy against sulphonamides and pyrimethamine (Doliwa *et al*., 2013*a*, 2013*b*). Continuous efforts have been made to develop drugs for the treatment of toxoplasmosis. However, drug development is an expensive and lengthy process (Hoelder *et al*., 2012). In an attempt to accelerate the process of drug discovery, older drugs are being tested and developed for new activities.

Lumefantrine, previously named benflumetol (a fluorene derivative belonging to the aminoalcohol class), is an antimalarial drug synthesized in the 1970s in China, which action mechanism is unclear (Olliaro and Trigg, 1995). Lumefantrine, which exhibits potent antimalarial activities, with a half-life of 3–5 days in malaria patients (Ezzet *et al*., 1998), can eliminate the *Plasmodium* parasites that remain in the blood following exposure to a fast-acting agent, such as artemisinin, thereby preventing recrudescence (Richard *et al*., 2014). Lumefantrine was widely used to treat different types of *Plasmodium*, which was assessed the interaction against 13 *Plasmodium falciparum* strains by isotopic test *in vitro* (Dormoi *et al*., 2014). A report about lumefantrine against 61 fresh clinical isolates of *P. falciparum* in Cameroon showed that lumefantrine possessed high activity *in vitro* compared with mefloquine, which were in agreement with the promising results of preliminary clinical trials (Basco *et al*., 1998). Lumefantrine also showed a good therapeutic effect on treating *Plasmodium berghei*, a developed lipidic system of lumefantrine exhibited excellent anti-*P. berghei* activity with 100% survival in male Swiss mice (Patil *et al*., 2013). In addition, lumefantrine is used to treat apicomplexans such as *Theileria equi* and *Babesia caballi* recently (Maji *et al*., 2019). As the first-line treatment of uncomplicated malaria caused by *P. falciparum* (WHO, 2010), lumefantrine was always combined with other agents, such as artemisinin, cepharanthine and atorvastatin (Desgrouas *et al*., 2014; Dormoi *et al*., 2014). In Guyana, the combination of lumefantrine and artemisinin has shown a better treatment effect for *Plasmodium vivax* than using lumefantrine or artemisinin alone. Chemotherapy drugs with well-matched pharmacokinetics are usually combined to improve treatment effect, and a combination of anti-malarial drugs usually associates a drug with a short elimination half-life and a drug with a long elimination half-life (Dormoi *et al*., 2014). Artemisinin is a kind of rapidly cidal antimalarial but with a high recurrence rate, whereas lumefantrine eliminates parasites more thoroughly and lasts for long time but the effect is slow (White *et al*., 1999), so the combination of these two drugs is often used in clinical malaria treatments (Eibach *et al*., 2012; Dormoi *et al*., 2014). Both *T. gondii* and *P. falciparum* are apicomplexan protozoa, however, the effect of lumefantrine on *T. gondii* has never been studied. There is an urgent need for the discovery of new anti-*T. gondii* drugs with high potency and less or no side-effects. Therefore, the aim of this study was to evaluate the activity of lumefantrine against *T. gondii* using cell culture and mice infected with *T. gondii* (RH strain) as *in vitro* and *in vivo* experimental models, respectively.

## Materials and methods

### Ethical standards

Experiments were performed using female Kunming (KM) mice (6–8 weeks old) obtained from Liaoning Changsheng Biotechnology Company, China. All animals were handled in strict accordance with good animal practice according to the Animal Ethics Procedures and Guidelines of the People's Republic of China, and the study was approved by the Animal Ethics Committee of Shenyang Agricultural University (Permit no. SYXK2011-0001), and all efforts were made to minimize suffering.

### Cells and parasites

Cells were cultured in 25 cm^2^ culture flasks in DMEM medium (Macgene, China) supplemented with 100 U mL^−1^ penicillin, 100 *μ*g mL^−1^ streptomycin (Macgene, China) and 10% heat-inactivated foetal bovine serum (FBS) (BI, Israel) at 37°C under a 5% CO_2_ atmosphere. *Toxoplasma gondii* tachyzoites (RH strain) were maintained in Vero cells cultured in DMEM medium supplemented with penicillin, streptomycin and 2% FBS at 37°C and 5% CO_2_.

### Cytotoxicity assay

Cytotoxicity of sulphadiazine and lumefantrine (Sigma, USA) to Vero cells was evaluated by the methyl thiazolyl tetrazolium (MTT) assay (Chen *et al*., 2008; Kavitha *et al*., 2010). Vero cells (2 × 10^5^) were seeded in 96-well plates and cultured in 10% FBS-DMEM for 12 h to obtain a monolayer. Vero cell monolayers were washed and directly subjected to lumefantrine (dilution from 50 to 1.563 *μ*g L^−1^) or sulphadiazine (dilution from 500 to 15.625 mg L^−1^, from 100 to 3.125 mg L^−1^ and from 30 to 0.9375 mg L^−1^, respectively), which were diluted with 10% FBS-DMEM. The Vero cells were subsequently cultured for 24 and 48 h. As a control, Vero cells were treated with 200 *μ*L 10% FBS-DMEM (blank control/DMEM group) and 20 *μ*L dimethyl sulphoxide (DMSO) (1 *μ*L mL^−1^) (Sigma, USA) together with 180 *μ*L 10% FBS-DMEM (solvent control/DMSO group). Supernatants were removed after culturing for 24 or 48 h, and the plates were washed twice by using phosphate-buffered saline (PBS) and pulsed by adding 10 *μ*L of MTT (Solarbio, China) together with 90 *μ*L 10% FBS-DMEM for 4 h under the same culture conditions. The supernatants were removed gently with pipettes and 110 *μ*L formazan was added to each well. The plates were vibrated on a low-speed oscillator, and optical density (OD) was measured at 490 nm by using a microplate reader after 30 min (Tecan, Switzerland).

### Proliferation assay *in vitro*

The anti-proliferation effect of lumefantrine on *T. gondii* was also detected using the MTT assay. Vero cell monolayers in 96-well plates were infected with 1 × 10^6^ fresh RH tachyzoites per well and incubated for 2 h at 37°C. Then, the Vero cell monolayers were washed twice with PBS to remove extracellular tachyzoites and incubated with DMEM (2% FBS) containing different concentrations of lumefantrine (50, 9.375 or 1.563 *μ*g L^−1^) for 24 and 48 h. The sulphadiazine (10 mg L^−1^) was added as a positive control. *Toxoplasma gondii*-infected Vero cells with DMEM only were used as a negative control. The MTT assay was carried out to evaluate parasite proliferation as previously described.

In addition, to further verify parasite proliferation, flow cytometry was conducted. Vero cell monolayers in six-well plates were infected with 1 × 10^6^ fresh RH tachyzoites per well and incubated for 2 h at 37°C. Then, the Vero cell monolayers were washed twice with PBS to remove extracellular tachyzoites and incubated with DMEM (2% FBS) containing different concentrations of lumefantrine and sulphadiazine for 24 h, respectively. Vero cells without RH tachyzoites were used as blank control and Vero cells seeded RH tachyzoites with DMEM only were used as negative control. After that, all the groups were digested by trypsin without EDTA at 37°C for 5 min, respectively, washed twice with PBS, stained with annexin V-labelled fluorescein 5-isothiocyanate (annexin V-FITC) (Biolegend, USA) and propidium iodide (Biolegend, USA), and incubated at room temperature for 10–15 min without light. Parasite proliferation was measured using a flow cytometer (BD, USA) (Hou *et al*., 2015).

### Effect of lumefantrine on mice infected by *T. gondii*

Seventy-two female mice (6–8 weeks) were divided into six treatment groups (12 mice per group). All the mice except for the blank control group (without *T. gondii* infection) were infected with fresh *T. gondii* (100 RH tachyzoites per mouse). After 24 h post-infection, the mice were given intragastric administration of sulphadiazine (10 mg L^−1^; dissolved in PBS) or lumefantrine (50, 9.375 or 1.563 *μ*g L^−1^; dissolved in PBS) every 2 days. Meanwhile, mice in both blank and negative groups (*T. gondii* infected-mice treated with PBS only) were injected intragastrically with the equal amounts of PBS. Mice were observed daily to record the death time and rate. All mice were humanely killed to collect blood at 11 days post-infection. Liver, heart, spleen and lung tissues were collected and stored in liquid nitrogen for RNA extraction.

### *Toxoplasma gondii* molecular detection in tissues

Tissue RNAs in different groups were extracted using Trizol (Invitrogen, USA), and the extracted RNAs were treated with DNase I (TaKaRa, China) to remove the genomic DNA. The mRNA was reverse transcribed from Oligo (dT) and used as templates for quantitative reverse transcription-polymerase chain reaction (RT-PCR). Specific primers (forward: TCCGGCTTGGCTGCTTT, reverse: TTCAATTCTCTCCGCCATCAC) were designed according to the gene sequence of *T. gondii* repeat region (AF146527.1), in which fragment was used to develop sensitive and specific PCR for diagnostic purposes (Homan *et al*., 2000; Pratama *et al*., 2015). Quantitative RT-PCR was performed on an ABI PRISM 7500 Real-Time PCR System (Applied Biosystems) and each reaction contained 10 *μ*L of 2 × TB Green *Premix E* *×* *Taq* (TaKaRa, China), 1 *μ*L of template cDNA, 6.6 *μ*L of distilled water, 0.4 *μ*L 50 × ROX Reference Dye II and 1 *μ*L each primer. The following amplification conditions were applied: 3 min at 95°C; 40 cycles of 95°C for 15 s (denaturation), 60°C for 40 s (annealing) and a dissociation step was added to confirm the amplification specificity for each gene. Experiment was repeated three times, and transcription levels were represented by the mean values of the three parallel experiments.

### Detection of interleukin (IL)-4, IL-10 and interferon gamma (IFN-*γ*)

The changes of IL-4, IL-10 and IFN-*γ* in mice treated with lumefantrine or sulphadiazine were evaluated using the cytokine ELISA (enzyme-linked immunosorbent assay) kits (Beyotime, China) according to the manufacturer's instructions. Sera of different treatment groups were collected at 11 days post-infection to detect the changes of cytokine levels through three independent experiments. Absorbance at 450 nm was measured by using a microplate reader (Tecan, Switzerland).

### Statistical analysis

Data were analysed using SPSS (ver18.0) computer software (SPSS for Windows, SPSS Inc., 2009). All values are expressed as mean ± s.d. Statistical analysis was performed using analysis of variance. *P* values less than 0.05 were considered statistically significant.

## Results

### Cytotoxicity activity

The MTT assay revealed that different concentrations of both lumefantrine and sulphadiazine had no cytotoxicity compared with the blank control (Supplementary Figs S1a and b). After calculation, the CC_50_ (50% cytotoxicity concentration) of lumefantrine was 4.75 × 10^8^ *μ*g L^−1^ at 24 h and 1.75 × 10^5^ *μ*g L^−1^ at 48 h. Thus, different concentrations of lumefantrine (high 50 *μ*g L^−1^, medium 9.375 *μ*g L^−1^ and low 1.563 *μ*g L^−1^) and sulphadiazine (10 mg L^−1^) were used to carry out further experiments against *T. gondii in vitro*.

### Anti-proliferation activity

**Fig. 1.**
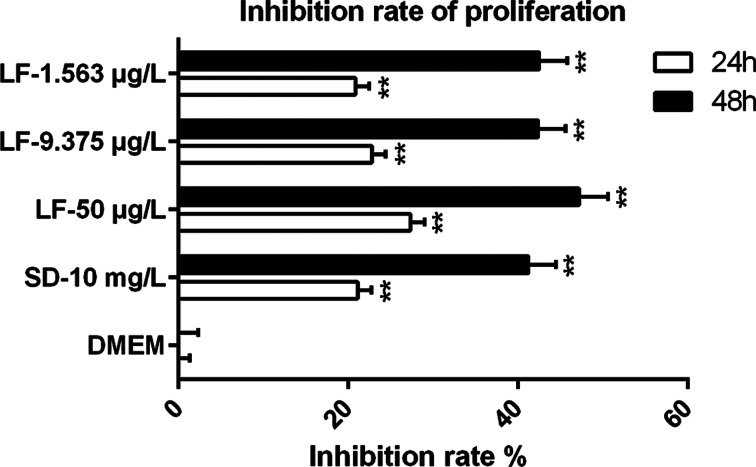
Effects of lumefantrine and sulphadiazine on *Toxoplasma gondii* proliferation. After 2 h pre-treatment of tachyzoites, Vero cells were separately treated with lumefantrine or sulphadiazine for other 24 and 48 h, respectively. Tachyzoites treated with DMEM only were defined as the negative control and those treated with sulphadiazine (10 mg L^−1^) as the positive control. The inhibition rates of *T. gondii* proliferation were calculated by the formula: (Group treatments OD − Group DMEM OD)/Group DMEM OD. Data represent mean ± s.d. of three independent experiments performed in triplicate. Significantly different from the negative control (compared with DMEM group, **P ≤ 0.01, *P ≤ 0.05).

Further evaluation of the ability of lumefantrine and sulphadiazine to inhibit the intracellular tachyzoite proliferation within Vero cells was examined using the MTT assay at 24 and 48 h post-treatment (Fig. 1). The absorbance could represent the number of living Vero cells, as parasites will damage living Vero cells when proliferation and invasion, therefore, the absorbance can reflect the inhibition effect of drugs against parasites indirectly. The IC_50_ (50% antiparasitic concentration) of lumefantrine against *T. gondii* proliferation was 139 *μ*g L^−1^ at 24 h and 51.48 *μ*g L^−1^ at 48 h. This was an indication that lumefantrine could significantly inhibit tachyzoite proliferation compared with the DMEM group (*P* ≤ 0.01).

**Fig. 2.**
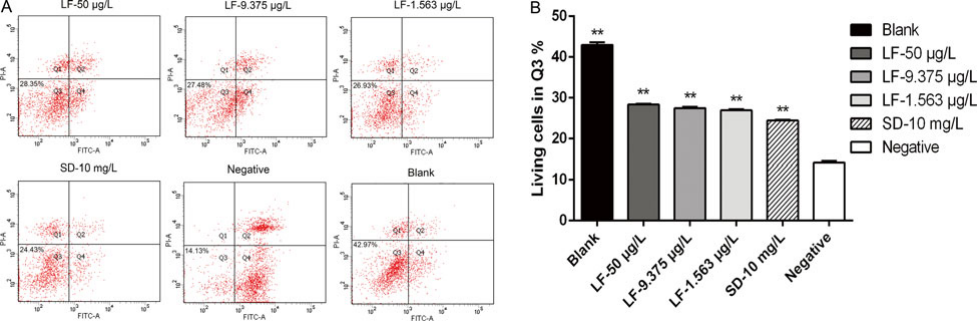
Lumefantrine inhibition of proliferation of *T. gondii* at 24 h post-treatment by flow cytometry. Tachyzoites were treated with lumefantrine for 24 h. Vero cells treated with DMEM only were defined as the blank control, and tachyzoites treated with DMEM only were defined as the negative control. As a positive control, tachyzoites were treated with sulphadiazine. Samples were stained with annexin V-FITC and propidium iodide, and the percentage of Vero cells in each group was determined by FCM. (a) Results of lumefantrine inhibit proliferation of *T. gondii* by FCM; (b) histogram based on the number of living Vero cells in the Q3 quadrant by FCM. Data represent mean ± s.d. of three independent experiments performed in triplicate. Significantly different from the negative control (compared with negative group, **P ≤ 0.01, *P ≤ 0.05).

The anti-proliferation activity of lumefantrine was further examined using flow cytometry. Samples were stained with annexin V-FITC and propidium iodide after treatment with lumefantrine or sulphadiazine for 24 h. Different quadrants represent different states of the Vero cells (Q1: necrotic and damaged Vero cells; Q2: late apoptotic Vero cells; Q3: living Vero cells; Q4: early apoptotic Vero cells). After *T. gondii* invasion and proliferation, they will bring some damage to Vero cells, so the purpose of flow cytometry was used to detect the number of living Vero cells in Q3 quadrant, which can reflect the drug anti-parasite effect indirectly. The more living Vero cells in Q3 quadrant reflect the better effect of lumefantrine on anti-parasite (Fig. 2a). These results showed that different concentrations of lumefantrine could inhibit the proliferation of *T. gondii* (*P* ≤ 0.01) by flow cytometry (Figs 2a and b).

### Survival rate of acutely infected mice treated with lumefantrine

**Fig. 3.**
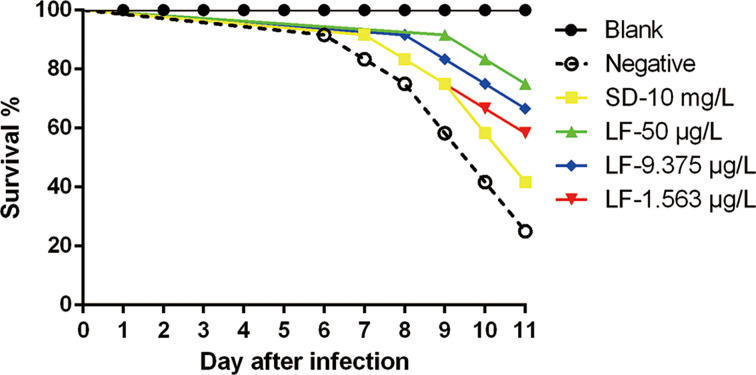
Effect of lumefantrine on the survival rate of acutely infected mice. All the mice were infected with 100 *T. gondii* tachyzoites and then treated with lumefantrine (50, 9.375 or 1.563 *μ*g L^−1^), a positive control (10 mg L^−1^ sulphadiazine) and PBS (negative control) every 2 days for 10 days. The uninfected control mice were served as blank group, and *T. gondii* infected-mice treated with PBS only were as the negative group. Mice were observed daily, and the survival rate was recorded for 11 days post-infection.

Mice were observed daily, and the survival rate was recorded for 11 days post-infection. *Toxoplasma gondii* infected-mice treated with PBS died at 6 days post-treatment. However, mice treated with 50, 9.375 or 1.563 *μ*g L^−1^ lumefantrine died at day 9, 8 and 7 post-treatment, respectively. The positive group (sulphadiazine group) died at day 7 post-treatment. After 11 days, 75, 66.7 and 58.3% of mice treated with 50, 9.375 and 1.563 *μ*g L^−1^ lumefantrine, respectively had survived, whereas only 41.7% living mice treated with 10 mg L^−1^ sulphadiazine had survived (Fig. 3).

### Parasite load in mice tissues

**Fig. 4.**
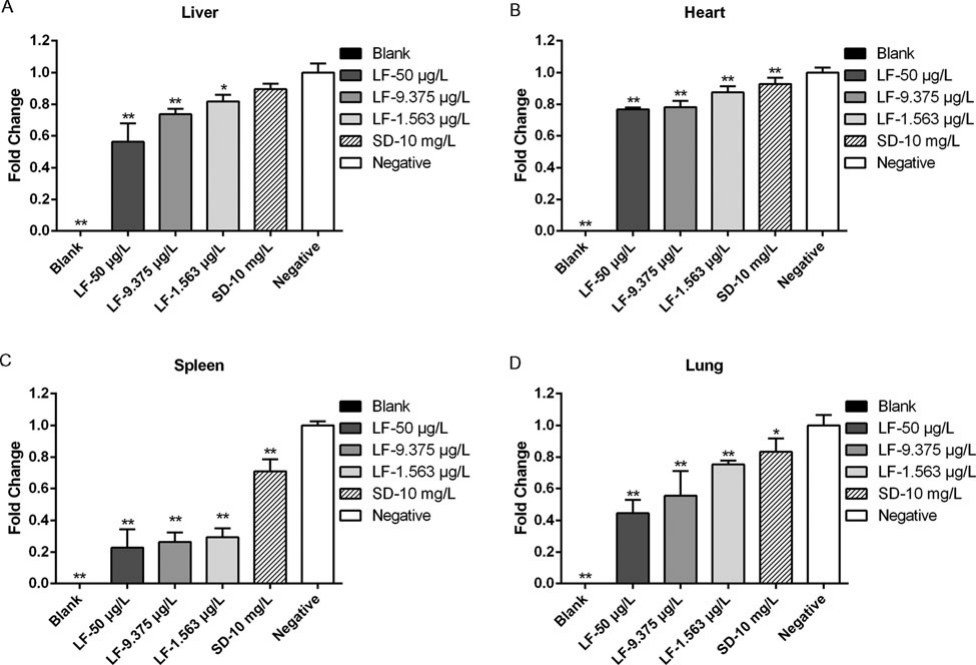
Parasite burden in tissues from the acutely infected mice. Mice were challenged intraperitoneally with 100 *T. gondii* tachyzoites, treated with lumefantrine (50, 9.375 or 1.563 *μ*g L^−1^), a positive drug (10 mg L^−1^ sulphadiazine) and PBS (negative control) every 2 days for 10 days. The uninfected control mice were served as blank group, and *T. gondii* infected-mice treated with PBS only were as the negative group. The parasite loads in the liver, heart, spleen and lung tissues of the infected mice were isolated and homogenized. Total RNA was isolated, and the *T. gondii* repeat region was detected by qPCR. The quantified parasite loads in the tissues of mice are presented as the fold change of −log_10_ values of the numbers of tachyzoites per 20 mg of tissues. Data represent mean ± s.d. of three independent experiments performed in triplicate. Significantly different from the negative control (compared with negative group, **P ≤ 0.01, *P ≤ 0.05).

To evaluate the parasite load in the mice after lumefantrine treatment, liver, heart, spleen and lung samples from infected mice were examined by qPCR, and the results are shown in Fig. 4. Treatment with different concentrations of lumefantrine significantly reduced the parasite load in the liver, heart, spleen and lung tissues compared to the negative control (PBS treated only) (*P* ≤ 0.01; *P* ≤ 0.05). The parasite load in different tissues except the liver was also reduced in the positive control group (sulphadiazine group).

### Regulation of cytokine levels by lumefantrine in mice infected by *T. gondii*

**Fig. 5.**
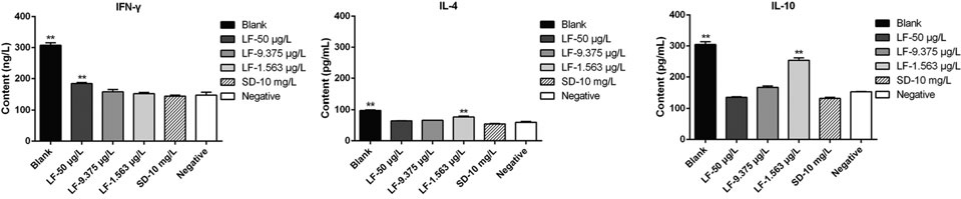
Lumefantrine regulates the change of cytokines. Mice were challenged intraperitoneally with 100 *T. gondii* tachyzoites and treated with lumefantrine (50, 9.375 or 1.563 *μ*g L^−1^), a positive drug (10 mg L^−1^ sulphadiazine) and PBS (negative control) every 2 days for 10 days. The uninfected control mice were served as blank group, and *T. gondii* infected-mice treated with PBS only were as the negative group. Sera of infected mice were collected at 11 days post-infection, and the cytokine levels were detected using a microplate reader. Cytokine levels were expressed as ng L^−1^ or pg mL^−1^. Three independent experiments were performed, and data are presented as mean ± s.d. Significantly different from the negative control (compared with negative group, **P ≤ 0.01, *P ≤ 0.05).

In order to determine whether lumefantrine treatment enhances Th1 or Th2 cytokine response, IFN-*γ*, IL-4 and IL-10 levels in the serum of mice were determined (Fig. 5). Significantly higher levels of IFN-*γ* were observed in mice treated with a high concentration lumefantrine compared to the negative control group (*P* ≤ 0.01), which indicated that high concentration lumefantrine could stimulate the hosts to produce IFN-*γ* to eliminate *T. gondii*. Meanwhile, IL-4 and IL-10 were significantly produced in mice treated with a low concentration lumefantrine compared to the negative control group (*P* ≤ 0.01). The results showed that lumefantrine could adjust the cytokines in hosts to eliminate parasites through the change of drug concentrations.

## Discussion

Lumefantrine has been shown to have a prominent inhibition effect on *P. vivax* (sexual and asexual stages), *P. falciparum*, *P. berghei*, *T. equi* and *B. caballi* (Eibach *et al*., 2012; Patil *et al*., 2013; Gimode *et al*., 2015; WorldWide Antimalarial Resistance Network (WWARN) Lumefantrine PK/PD Study Group, 2015; Maji *et al*., 2019). The terminal elimination half-life of a drug is an important determinant of the propensity for an anti-malarial drug to select for resistance. Therefore, the mismatch between the short-acting artemisinin derivative and the long-acting partner drug provides selection pressure for emergence of resistant parasites, since one drug is rapidly eliminated and the other drug persists alone (Gimode *et al*., 2015). Lumefantrine is a longer-acting drug, and confers protection against recrudescence following malaria infection (Kokwaro *et al*., 2007). At present, artemether/lumefantrine (AL) is the only fixed-dose artemisinin-based combination therapy recommended and pre-qualified by the World Health Organization (WHO) for the treatment of uncomplicated malaria caused by *P. falciparum*. It has been shown to be effective both in sub-Saharan Africa and in areas with multi-drug resistant *P. falciparum* in southeast Asia (Kokwaro *et al*., 2007). It is currently recommended as first-line treatment for uncomplicated malaria in several countries. *Toxoplasma gondii* is an apicomplexan protozoa, which is like *Plasmodium*. Thus, we can infer that lumefantrine may act against apicomplexan parasites. It is necessary to explore whether lumefantrine has anti-*T. gondii* activity *in vitro* and *in vivo*. Besides, studies that have shown successful treatment for toxoplasmosis patients are limited, indicating the urgent need to identify and develop new therapies (Adeyemi *et al*., 2018) and data about the inhibition of *T. gondii* using lumefantrine is not available. Therefore, in this study, we evaluated the effect of lumefantrine treatment on *T. gondii* infection *in vivo* and *in vitro*.

The results showed that lumefantrine demonstrated activity against *T. gondii* RH strain tachyzoites. Lumefantrine affects the intracellular of *T. gondii* tachyzoites in a concentration-dependent manner compared with the negative control (DMEM group) (*P* ≤ 0.01), as determined through *in vitro* anti-proliferation assays. Furthermore, lumefantrine showed low cytotoxicity in Vero cells and the findings are consistent with the previous report (Kokwaro *et al*., 2007), and the CC_50_ of lumefantrine for Vero cells was 3 417 266-fold higher than the IC_50_ against *T. gondii* at 24 h and 3399-fold higher at 48 h, which demonstrates that lumefantrine has a high therapeutic index and the use of lumefantrine has a wide safety range. In addition, compared with other recently described natural products, matrine (ME) (Zhang *et al*., 2016), ginkgolic acids (Choi *et al*., 2008) and other plant extracts (Sepulveda-Arias *et al*., 2014), the IC_50_ of lumefantrine was lower than those drugs, indicating that at the same concentration, the anti-*T. gondii* activity of lumefantrine was better than natural products, matrine (ME), and ginkgolic acids. Anti-proliferation assay showed that a 21.12% reduction at 24 h and a 41.2% reduction at 48 h post-treatment with lumefantrine were recorded (*P* ≤ 0.01), which was also verified by flow cytometry. Accordingly, we can conclude that lumefantrine presents a potent anti-*T. gondii* activity *in vitro*.

Based on the *in vitro* results above, we sought to determine whether lumefantrine exerts anti-*T. gondii* effects on acute infections *in vivo*. Thus, a mouse model was established by infecting mice with the virulent RH strain of *T. gondii*. Before that, we treated healthy mice with the same doses of lumefantrine, and all the mice survived. Evaluation of anti-*T. gondii* effects of lumefantrine on mice acutely infected by the RH strain of *T. gondii* revealed 75, 66.7 and 58.3% of mice treated with 50, 9.375 and 1.563 *μ*g L^−1^ lumefantrine, respectively had survived at 11 days post-treatment, and only 41.7% living mice treated with 10 mg L^−1^ sulphadiazine had survived. Furthermore, the parasite burdens in the liver, heart, spleen and lung after lumefantrine treatment were significantly decreased compared with those in the negative control group (PBS-treated only) (*P* ≤ 0.01; *P* ≤ 0.05), indicating that lumefantrine exerts an inhibitory effect on *T. gondii*, partially provides protection against death due to *T. gondii* infection, and reduces the parasite burden in the tissues of mice. Lumefantrine has a wide safety range and a small side-effect. High levels of Th1 (IFN-*γ*) and Th2 (IL-4 and IL-10) cytokines were detected in lumefantrine-treated mice. IFN-*γ* was the key cytokine in resistance against *T. gondii* infection (Dautu *et al*., 2007). IFN-*γ* can inhibit the proliferation of *T. gondii* in infected host cells through various mechanisms, including induction of the inhibitory protein guanamine 2,3-dioxygenase, inducible nitric oxide synthase, the effector proteins immunity-related GTPases and guanylate-binding proteins (Zheng *et al*., 2019*a*). In the current study, a significant increase in IFN-*γ* production in mice treated with a high-dose lumefantrine improved mouse survival (*P* ≤ 0.01). These results indicate that lumefantrine can trigger an increased IFN-*γ* production and contribute to the prevention of acute *T. gondii* infection. Meanwhile, an increase in IL-10 and IL-4 levels was also observed in mice, which received a low dose of lumefantrine (*P* ≤ 0.01). IL-10 has a central role in limiting inflammation and inhibiting CD4+ T cell-mediated severe immunopathology (Dupont *et al*., 2012), and IL-4 functions to enhance IFN-*γ* production in the late stage of infection (Zheng *et al*., 2019*b*).

Lumefantrine is a kind of antimalarial drug with a long half-life period, the mechanism of action and resistance mechanism of lumefantrine is still not clear. It belongs to aromatic cyclic methanol, and it's actually in the same class as quinine, which is also an important antimalarial drug (Xi, 2006). Quinine can bind to the DNA of the malaria parasite, forming complex and inhibiting DNA replication and RNA transcription, thus inhibit the protozoan protein synthesis (Xi, 2006). Based on these results in this study, we speculate that lumefantrine may be used to treat toxoplasmosis patients or people who suffer combination infections of *T. gondii* and *Plasmodium* clinically, meanwhile, the combination of artemether and lumefantrine may play a better effect for treating patients with *T. gondii* infections. Thus, more experiments on the protective and therapeutic mechanisms of lumefantrine should be undertaken to understand the effects of lumefantrine only or artemether/lumefantrine combination on *T. gondii* tachyzoites and bradyzoites or on different *T. gondii* types.

## Supplementary material

For supplementary material accompanying this paper visit https://doi.org/10.1017/S0031182020002036. click here to view supplementary material
