# Supplementary figures and images for: Determination of lumefantrine as an effective drug against Toxoplasma gondii infection – in vitro and in vivo study
Source: Parasitology. 2020 Oct 22;148(1):122–8. doi: 10.1017/S0031182020002036 (PMC7808861; doi:10.1017/S0031182020002036)

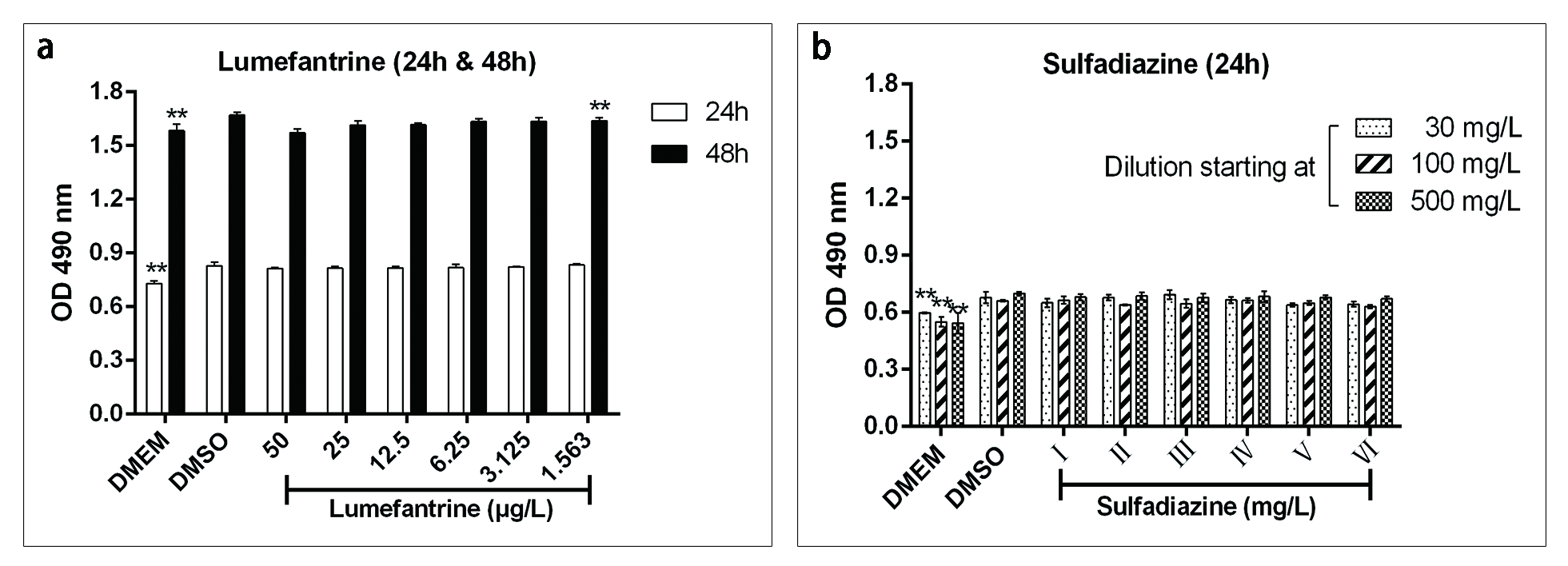

Supplement: Supplementary file 1 [file S0031182020002036sup001.tif]
